# Prevalence of Mental Health Diagnoses in Commercially Insured Children and Adolescents in the US Before and During the COVID-19 Pandemic

**DOI:** 10.1001/jamanetworkopen.2023.14415

**Published:** 2023-05-22

**Authors:** Loreen Straub, Brian T. Bateman, Seanna Vine, Krista F. Huybrechts

**Affiliations:** 1 Division of Pharmacoepidemiology and Pharmacoeconomics, Brigham and Women’s Hospital and Harvard Medical School, Boston, Massachusetts; 2 Department of Anesthesiology, Perioperative and Pain Medicine, Stanford University School of Medicine, Stanford, California

## Abstract

This cross-sectional study examines trends in the prevalence of various mental health diagnoses in children and adolescents in the US, stratified by age and sex, before and during the COVID-19 pandemic.

## Introduction

In 2021, several pediatric health organizations declared a state of emergency due to the perception that mental health (MH) conditions were increasing among youths, seemingly exacerbated by the COVID-19 pandemic.^1^ The COVID-19 pandemic caused disruptions in daily life, social isolation, family economic burden, increased social media engagement, and reduced access to care, all of which might have negative associations with MH. To our knowledge, no nationwide data evaluating trends in pediatric MH diagnoses during the pandemic, stratified by age and sex, are currently available for the US.^2,3,4,5^

## Methods

This cross-sectional study was approved by the institutional review board of Brigham and Women’s Hospital, with a waiver of informed consent because the data were deidentified. This study follows the Strengthening the Reporting of Observational Studies in Epidemiology (STROBE) reporting guideline.

**Figure 1.**
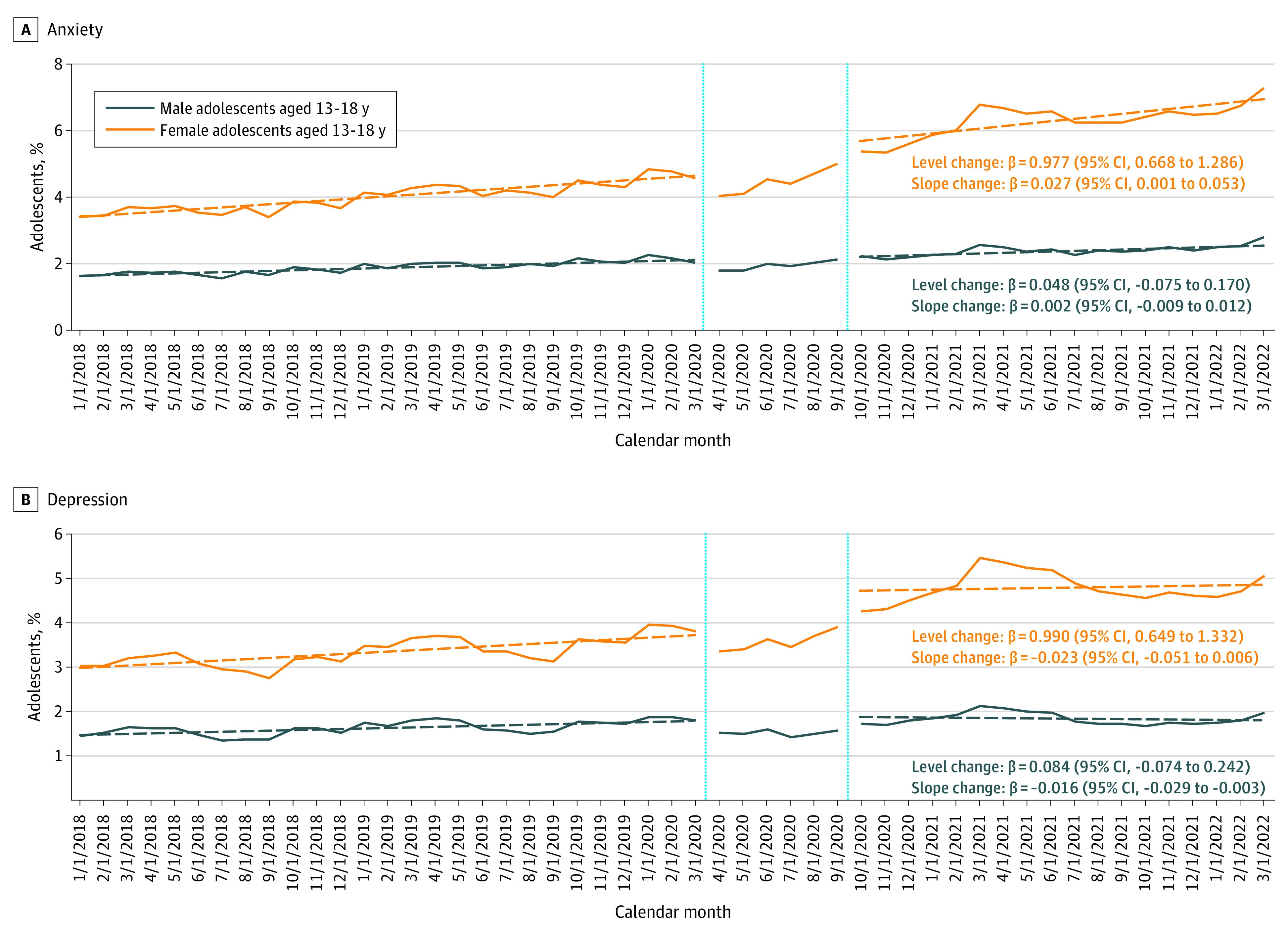
Monthly Percentage of Male and Female Adolescents Aged 13 to 18 Years With Mental Health Diagnoses of Anxiety or Depression Between January 2018 and March 2022 The β-coefficients with corresponding 95% CIs in parentheses represent the change in level and slope in percentage of adolescents with the respective mental health conditions. Solid lines represent the observed trends. Dashed lines represent the estimated linear trends in the respective period. The vertical lines divide the assessment period into 3 periods: the prepandemic period (January 2018 to March 2020), the early period of the pandemic when school closure was common (April 2020 to September 2020), and the recent period of the pandemic after schools started to reopen (October 2020 to March 2022). Different y-axis scales were chosen to account for variations in prevalence across mental health diagnostic groups. The monthly proportion of 13- to 18-year-old male and female adolescents with mental health diagnoses was assessed according to the presence of at least 1 outpatient or inpatient mental health diagnosis of interest among all youths enrolled in the respective month.

**Figure 2.**
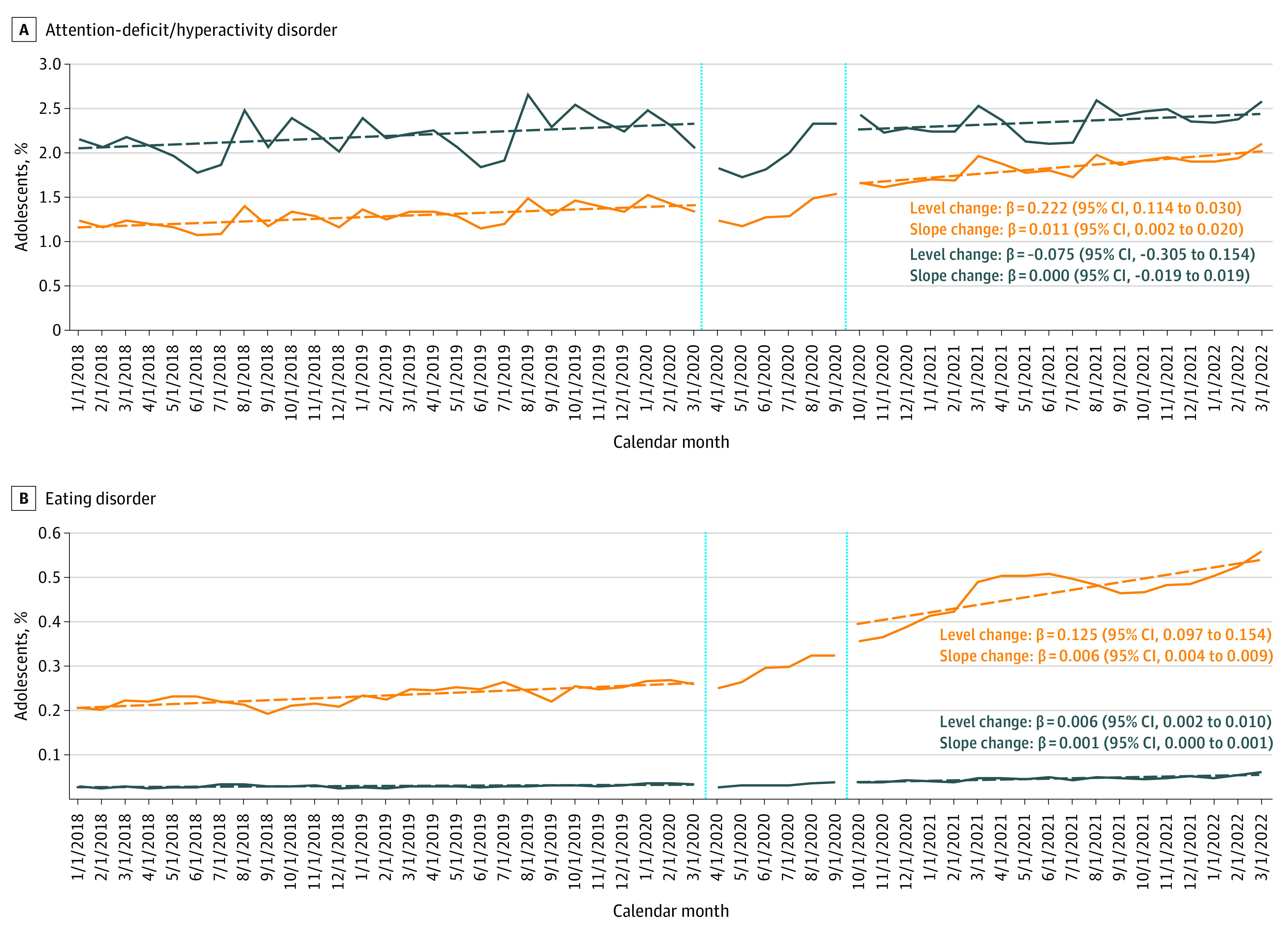
Monthly Percentage of Male and Female Adolescents Aged 13 to 18 Years With Mental Health Diagnoses of Attention-Deficit/Hyperactivity Disorder and Eating Disorder Between January 2018 and March 2022 The β coefficients with corresponding 95% CIs in parentheses represent the change in level and slope in percentage of adolescents with the respective mental health conditions. Solid lines represent the observed trends. Dashed lines represent the estimated linear trends in the respective period. The vertical lines divide the assessment period into 3 periods: the prepandemic period (January 2018 to March 2020), the early period of the pandemic when school closure was common (April to September 2020), and the recent period of the pandemic after schools started to reopen (October 2020 to March 2022). Different y-axis scales were chosen to account for variations in prevalence across mental health diagnostic groups. The monthly proportion of 13- to 18-year-old male and female adolescents with mental health diagnoses was assessed according to the presence of at least 1 outpatient or inpatient mental health diagnosis of interest among all youths enrolled in the respective month.

Using a geographically diverse commercial health care claims database (Optum deidentified Clinformatics Data Mart Database; eAppendix in Supplement 1), we assessed the monthly proportion of youths, stratified by age and sex, who received an MH diagnosis between January 2018 and March 2022. We considered the following commonly diagnosed groups of MH conditions in pediatric patients aged 6 to 18 years in our cohort: anxiety disorders, attention-deficit/hyperactivity disorder (ADHD), depression, and eating disorders (eAppendix and eTable in Supplement 1). We assessed 3 time periods of the COVID-19 pandemic: the prepandemic period (January 2018 to March 2020), the early pandemic period when school closure was common (April 2020 to September 2020), and the recent pandemic period after schools started to reopen (October 2020 to March 2022). Interrupted time series analyses were conducted to compare the prevalence and trends of each diagnosed MH condition in the prepandemic vs the recent pandemic period (Figure 1 and Figure 2). Data from the early pandemic period—the transition period when the pandemic was in a state of flux—were not included in interrupted time series analyses to allow time for the potential impact of the pandemic to manifest itself. All data were analyzed using SAS statistical software version 9.4 (SAS Institute), and 2-sided 95% CIs were calculated. Data analysis was conducted from October 2022 to March 2023.

## Results

On average, approximately 1.7 million youths contributed data to each calendar month. Of those participants, on average, 440 722 were 6- to 12-year-old female children (average proportion [SD] per month, 25.3% [0.6%]), 410 373 were 13- to 18-year-old female adolescents (23.6% [0.6%]), 461 331 were 6- to 12-year-old male children (26.5% [0.6%]), and 426 358 were 13- to 18-year-old male adolescents (24.5% [0.7%]).

Among 13- to 18-year-old female adolescents, there was an immediate increase in the prevalence of all 4 diagnosed MH conditions in the recent pandemic period (level change) (Figure 1 and Figure 2). Except for depression, the prevalence of MH diagnoses in this group increased at a faster rate during the pandemic than before the pandemic (slope change). Most strikingly, the prevalence of diagnosed eating disorders more than doubled during the pandemic (from 1065 adolescents [0.26%] in March 2020 to 1399 adolescents [0.36%] in October 2020 and 2058 adolescents [0.56%] in March 2022).

Although the prevalence of eating disorders was considerably lower in 13- to 18-year-old male adolescents, trends were similar when compared with 13- to 18-year-old female adolescents (from 114 male adolescents [0.03%] in March 2020 to 234 male adolescents [0.06%] in March 2022). Prevalence changes before vs during the pandemic were not observed for other MH diagnoses among 13- to 18-year-old male adolescents.

Among 6- to 12-year-old children, the prevalence of all diagnosed MH conditions, except for ADHD, was lower when compared to adolescents (not shown). The prevalence of eating disorders showed trends similar to those observed among 13- to 18-year-old adolescents, with relatively stable estimates in the prepandemic period and upward trends in the recent pandemic period (from 115 female children [0.03%] to 189 female children [0.05%]; and from 65 male children [0.01%] to 118 male children [0.03%]). Among 6- to 12-year-old female children, prevalence changes for other MH diagnoses were similar to but far less pronounced than those for females adolescents. No trends for other MH diagnoses were observed among 6- to 12-year-old male children.

## Discussion

In summary, this cross-sectional study suggests that trends in MH diagnoses differed greatly by age and sex over the course of the COVID-19 pandemic. Female youth, especially female adolescents, represented the most vulnerable population with regard to marked increases in the prevalence of MH diagnoses during the pandemic, the most pronounced being the prevalence of eating disorders.

This study is limited to commercially insured youths; thus, no inferences can be made about diagnosis patterns in publicly insured or uninsured youths. Due to heterogeneous sampling, the cohort might not be completely representative of all commercially insured youths across the US.^6^ Furthermore, recorded MH diagnoses might not reflect the true MH status of the youths in the study. These findings highlight the urgency to identify the underlying factors associated with the increase in MH diagnoses in female adolescents (eg, social isolation or accelerated reliance on social media), so that targeted mitigation strategies can be developed to reverse the alarming trend which has continued several years into the COVID-19 pandemic.

## Notes

Supplement 1. **eAppendix.** Supplemental Methods **eTable.** Definition of Selected Mental Health Conditions Based on Recorded Diagnostic Codes
eReference

Supplement 2.
Data Sharing Statement
