# Supplementary material for: Prevalence of Mental Health Diagnoses in Commercially Insured Children and Adolescents in the US Before and During the COVID-19 Pandemic
Source: JAMA Netw Open. 2023 May 22;6(5):e2314415. doi: 10.1001/jamanetworkopen.2023.14415 (PMC10203892; doi:10.1001/jamanetworkopen.2023.14415)
Supplement: Supplement 1. — eAppendix. Supplemental Methods eTable. Definition of Selected Mental Health Conditions Based on Recorded Diagnostic Codes eReference [file jamanetwopen-e2314415-s001.pdf]

Supplemental Online Content

Straub L, Bateman BT, Vine S, Huybrechts KF. Prevalence of mental health diagnoses in commercially insured children and adolescents in the US before and during the COVID-19 pandemic. *JAMA Netw Open*. 2023;6(5):e2314415. doi:10.1001/jamanetworkopen.2023.14415

eAppendix. Supplemental Methods
eTable. Definition of Selected Mental Health Conditions Based on Recorded Diagnostic Codes
eReference

This supplemental material has been provided by the authors to give readers additional information about their work.

© 2023 Straub L et al. *JAMA Network Open.*

eAppendix. Supplemental Methods

Description of Optum Clinformatics Data Mart database

Optum's de-identified Clinformatics® Data Mart Database (CDM) database comprises a large, geographically diverse population of health insurance beneficiaries enrolled in commercial UnitedHealth Group-affiliated and Medicare Advantage health plans, spanning all 50 US states and Washington, DC. CDM data are updated quarterly and available with about a 6-month lag time. About one quarter of the cohort included in the CDM data is less than 18 years of age and duration of observation of enrollees included in CDM is ~2.6 years on average.

Definition of and rationale for selected mental health conditions

<u>Definition:</u>

Presence of individual mental health conditions was determined based on ≥1 inpatient or outpatient ICD-10-CM diagnostic code of interest recorded in the patient's health insurance claims during the respective assessment period. The codes considered for each selected condition are listed in the table below.

<u>Justification for assessment of four selected mental health conditions:</u>

We selected the three most commonly diagnosed mental health conditions in our cohort, which were: attention deficit/hyperactivity disorder, anxiety disorder and depression. We further included diagnosis of eating disorders as the 4th condition of interest because a previous publication – which focused on trends in pediatric primary care visits in Massachusetts for selected mental health categories – reported a striking increase in eating disorder visits during the pandemic period.[1] Since these data were not stratified by sex & age and included only information from one state, we sought to assess whether the same increase was seen in the data spanning all US States and Washington, DC. While eating disorders are not as common as other mental health conditions in younger children, they were the 4th most common diagnostic group in 13-18-year-old females in the more recent period in our cohort.

eTable Definition of selected mental health conditions based on recorded diagnostic codes

**Mental Health Condition**	**ICD-10-CM Codes**
Attention deficit/hyperactivity disorder	F90.*
Anxiety disorder	F06.4, F40.*, F41.*, F42.*, F43.0, F43.1*, F43.22, F93.8
Depression	F32.* (excluding F32.8*), F33.* (excluding F33.8), F06.3*, F34.1, F43.21, F43.23
Eating disorder	F50.*

Abbreviations: ICD-10-CM, International Classification of Diseases, Tenth Revision, Clinical Modification.

eReference:

1. Bittner Gould J, Walter HJ, Bromberg J, Correa ET, Hatoun J, Vernacchio L. Impact of the Coronavirus Disease 2019 Pandemic on Mental Health Visits in Pediatric Primary Care. *Pediatrics*. Dec 01 2022;150(6)doi:10.1542/peds.2022-057176

© 2023 Straub L et al. *JAMA Network Open.*
