# Supplementary material for: Prevalence of Mental Health Diagnoses in Commercially Insured Children and Adolescents in the US Before and During the COVID-19 Pandemic
Source: JAMA Netw Open. 2023 May 22;6(5):e2314415. doi: 10.1001/jamanetworkopen.2023.14415 (PMC10203892; doi:10.1001/jamanetworkopen.2023.14415)
Supplement: Supplement 2. — Data Sharing Statement [file jamanetwopen-e2314415-s002.pdf]

Data Sharing Statement

Straub. Prevalence of Mental Health Diagnoses in Commercially Insured Children and Adolescents in the US Before and During the COVID-19 Pandemic. *JAMA Netw Open*. Published May 22, 2023. doi:10.1001/jamanetworkopen.2023.14415

Data
Data available: No
